# *Glissandra oviformis* n. sp.: a novel predatory flagellate illuminates the character evolution within the eukaryotic clade CRuMs

**DOI:** 10.1098/rsob.250057

**Published:** 2025-06-04

**Authors:** Euki Yazaki, Ryo Harada, Ryu Isogai, Kohei Bamba, Ken-ichiro Ishida, Yuji Inagaki, Takashi Shiratori

**Affiliations:** ^1^ National Agriculture and Food Research Organization Advanced Analysis Center, Tsukuba, Ibaraki Prefecture, Japan; ^2^ iTEMS, RIKEN, Wako, Saitama Prefecture, Japan; ^3^ Department of Biochemistry and Molecular Biology, Dalhousie University, Halifax, Nova Scotia, Canada; ^4^ Graduate School of Science and Technology, University of Tsukuba, Tsukuba, Ibaraki Prefecture, Japan; ^5^ Institute of Life and Environmental Sciences, University of Tsukuba, Tsukuba, Ibaraki Prefecture, Japan; ^6^ Center for Computational Sciences, University of Tsukuba, Tsukuba, Ibaraki Prefecture, Japan

**Keywords:** CRuMs, protists with uncertain phylogenetic affiliations, phylogenomics, ultrastructures

## Abstract

Culturing protists offers a powerful approach to exploring eukaryotic diversity, especially for deep-branching lineages. In this study, we cultured and described a novel protist species, named *Glissandra oviformis* n sp. within the poorly studied and unclassified genus *Glissandra*. While an SSU rDNA gene phylogeny failed to resolve its phylogenetic placement in the eukaryotic tree, a phylogenomic analysis of 340 proteins indicated *G. oviformis* as a member of the CRuMs clade. Prior to this study, this clade consisted of diverse heterotrophic amoeba and flagellates, and lacked clear synapomorphies. Ultrastructural observations revealed that *G. oviformis* shares the characteristics with some CRuMs members, including the pellicle underlying the plasma membrane and an internal sleeve surrounding the central pair of the axoneme at the flagellar transitional region. Our findings suggest potential shared characteristics and synapomorphies for CRuMs and contribute to a deeper understanding of the character evolution within this clade.

**Figure:**
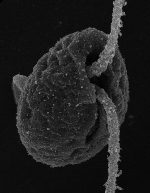

## 1. Introduction

Protists, eukaryotes excluding animals, land plants and fungi, are a major component of the eukaryotic tree of life (eToL) and are remarkably diverse in their morphology and ecological roles [1,2]. However, compared to multicellular organisms, our understanding of unicellular protists remains limited due to their small size and difficulties in cultivation. Indeed, metabarcoding studies have highlighted the existence of a wide range of eukaryotic lineages, including protists, but many of these remain uncharacterized in natural environments [3,4]. The limited understanding of protists suggests that there are many opportunities for new discoveries. Indeed, new protistan taxa and lineages have continued to be found, even novel supergroup-level lineages [5,6]. Such discoveries are often based on identifying entirely novel protists or reinvestigating previously described protists with uncertain phylogenetic affiliations (PUPA). These PUPA were described but not assigned to any major eukaryotic lineage due to limited morphological or molecular data. Some PUPA have been placed within well-known supergroups upon reinvestigation [7–9], while others are revealed as distinct deep-branching lineages within the eToL [10,11]. The long history of protist taxonomy has accumulated a substantial list of PUPA in need of reinvestigation [1,12]. Their rediscovery and characterization are essential to understanding the diversity and evolution of eukaryotes precisely.

Recent phylogenetic analyses based on multiple proteins (phylogenomic analyses) have clarified the phylogenetic homes for several PUPA, such as *Microheliella*, Picozoa and *Telonema* [13–15]. Intriguingly, morphologically dissimilar PUPA were occasionally shown to form unexpected monophyletic groups [5,16]. One prominent example is CRuMs, one of the major eukaryotic clades recently recognized [16]. CRuMs was formed by three groups of PUPA: the swimming predatory flagellates (diphylleids), the non-flagellated filose amoebae (rigifilids) and the marine, tiny gliding flagellates (*Mantamonas*). Despite strong phylogenetic support for the monophyly of CRuMs, their shared characteristics and synapomorphies remain elusive.

In this study, we successfully established a culture of a new species of a previously unclassified protist genus *Glissandra*, described here as *G. oviformis* n. sp. A phylogenomic analysis placed *G. oviformis* as a novel lineage within member of CRuMs. Furthermore, through ultrastructural observations, we inferred the shared characteristics and synapomorphies of CRuMs. These findings provide new insights into this enigmatic group and highlight the significance of PUPA in advancing our understanding of eukaryotic diversity and evolution.

## 2. Material and methods

### 2.1. Sample collection and culture establishment

A seaweed (*Halimeda* sp.) was collected in Coral Lake, the Republic of Palau (7.2510° N, 134.3738° E) on 1 November 2013. The seaweed was washed with IMK medium (Nihon Pharmaceutical), and the resulting wash medium was incubated at 20℃ under a 14 h light and 10 h dark cycle for five weeks. A single cell of *G. oviformis* n. sp. was isolated by micropipetting and incubated with a culture of *Pedinomonas* sp. or *Bigelowiella natans* in IMK medium. The resulting two-member culture of *G. oviformis* with *Pedinomonas* sp. or *B. natans* was maintained at 20℃ under a 14 h light and 10 h dark cycle until April 2019.

### 2.2. Light microscopy

For light microscopic observation, cells of *G. oviformis* were observed on microscope slides using a Zeiss Axio imager A2 microscope (Zeiss) equipped with an Olympus DP71 CCD camera (Olympus).

### 2.3. Electron microscopy

For scanning electron microscopy, the cell suspension of *G. oviformis* was mounted on an 8.5 mm diameter glass SEM plate (Okenshoji) treated with 0.1% (w/v) poly l-lysine. Cells sticking on the plate were pre-fixed with 2.5% glutaraldehyde, 0.1% osmium tetroxide (OsO_4_) and 0.2 M sucrose in 0.2 M sodium cacodylate buffer (CB) (pH 7.2) for 30 min and then washed with 0.2 M CB three times. The plate was placed in 0.2 M CB with 1% OsO_4_ for 1 h and washed with 0.2 M CB 10 times. The plate was then placed in 0.2 M CB with 1% tannic acid for 30 min and washed with 0.2 M CB 10 times. Finally, the plate was placed in 0.2 M CB with 1% OsO_4_ for 1 h again. The plate was dehydrated in a series of 15−100% (v/v) ethanol. After dehydration, the plate was placed once in a 1:1 mixture of 100% ethanol and t-butyl alcohol, twice in 100% t-butyl alcohol and chilled in the freezer. The plate was freeze-dried using a VFD-21S (Shinku) freeze drier and then mounted on aluminium stubs using carbon paste. The specimen was sputter-coated with platinum–palladium using a Hitachi E-102 sputter-coating unit (Hitachi High-Technologies) and observed using a JSM-6360F field emission SEM (JEOL).

For transmission electron microscopy, the cells of *G. oviformis* were collected by centrifugation at 3000 *g* for 10 min. Cell pellets were treated in a mixture of 2% (w/v) glutaraldehyde, 0.2 M CB and 0.25 M sucrose for 1 h at room temperature for pre-fixation. Cells were then washed with 0.2 M CB three times. Cells were then post-fixed with 1% (v/v) OsO4 in 0.2 M CB, and dehydration was performed using a graded series of 30−100% ethanol (v/v). After dehydration, cells were placed in a 1 : 1 mixture of 100% ethanol and acetone, followed by pure acetone twice. Resin replacement was performed using a 1:1 mixture of acetone and Agar Low Viscosity Resin R1078 (Agar Scientific), followed by pure resin. The resin was polymerized by heating at 60°C for 12 h. Ultrathin sections were prepared on a Reichert Ultracut S ultramicrotome (Leica), double-stained with 2% (w/v) uranyl acetate and lead citrate, and observed using a Hitachi H-7650 electron microscope (Hitachi) equipped with a Veleta TEM CCD camera (Olympus).

### 2.4. DNA extraction and SSU rDNA PCR

The total DNA of the two-member culture was extracted from a cell pellet obtained by centrifuge using DNeasy plant mini kit (Qiagen Science), according to the manufacturer’s instructions. Small subunit ribosomal DNA (SSU rDNA) was amplified by PCR with forward primer 18F and reverse primer 18R [17]. Amplifications consisted of 30 cycles of denaturation at 94℃ for 30 s, annealing at 55℃ for 30 s and extension at 72℃ for 2 min. Amplified DNA fragments were purified after gel electrophoresis with a QIAquick Gel Extraction Kit (Qiagen Science) and then cloned into the p-GEM T-easy vector (Promega). The inserted DNA fragments were completely sequenced by a 3130 Genetic Analyzer (Applied Biosystems). The SSU rDNA sequence of *G. oviformis* is deposited as LC868286 in GenBank.

### 2.5. SSU rDNA phylogenetic analysis

The SSU rDNA sequence of *G. oviformis* was aligned with those of 190 phylogenetically diverse eukaryotes using MAFFT v. 7.520 [18]. After removing ambiguously aligned positions using BMGE v. 1.12 [19] with default settings, 1137 nucleotide positions remained. This alignment was subjected to the maximum likelihood (ML) phylogenetic analysis using IQ-TREE v. 2.2.2.6 [20] with the SYM+ I + R6 model (automatically determined by IQ-TREE’s ModelFinder) [21]. ML bootstrap percentage values (MLBPs) were obtained from 100 non-parametric bootstrap replicates. Additionally, the SSU rDNA alignment was analysed using Bayesian phylogenetic method using the CAT + GTR model with PhyloBayes-mpi v. 1.8a [22–24]. In this analysis, two MCMC runs were conducted for 100 000 cycles with a burn-in of 25 000 cycles. A consensus tree with branch lengths and Bayesian posterior probabilities (BPPs) was then calculated from the remaining trees.

### 2.6. RNA extraction and RNA-seq

The total RNA of the two-member culture was extracted from a cell pellet using the Trizol reagent (Life Technologies) following the manufacturer’s instructions. The cDNA library construction and paired-end sequencing (125 bp per read) with HiSeq2000 (Illumina) were performed at Eurofins Genomics. For *G. oviformi*s, 3.3 × 10^7^ paired-end 125 bp reads (8.2 Gb in total) were obtained and then performed quality filtering using Trimmomatic v. 0.39 [25] with the following parameters: LEADING = 30, TRAILING = 30, SLIDINGWINDOW = 4:25 and MINLEN = 20. The filtered reads were then mapped to the CDS of *B. natans* (Bigna1) [26] downloaded from EnsemblProtists (https://ftp.ensemblgenomes.ebi.ac.uk/pub/protists/release-59/fasta/bigelowiella_natans/cds/) using HISAT2 v. 2.2.1 [27]. Unmapped reads were assembled using Trinity v. 2.15.1 [28,29] to obtain unique contigs of 81 591. The contigs were translated into protein sequences using TransDecoder v. 5.7.1 (https://github.com/TransDecoder/TransDecoder), resulting in the identification of 376 986 protein sequences.

### 2.7. Phylogenomic analyses

To elucidate the phylogenetic position of *G. oviformis*, we prepared a phylogenomic alignment by updating an existing one comprising 351 proteins [30]. For each protein, we added homologous sequences retrieved by BLASTp (with an E-value cut-off of 10⁻^30^) from the newly generated *G. oviformis* protein sequences (see above), as well as sequences from the provorans, anaeramoebids and other organisms (total 50 species including *G. oviformis* added, described in electronic supplementary material, table S1). Individual single-protein alignments were generated using MAFFT v. 7.520 with the L-INS-i algorithm, followed by manual correction and automated exclusion of ambiguously aligned positions using BMGE v. 1.12.

Each single-protein alignment was subjected to a preliminary phylogenetic analysis using FastTree v. 2.1.11 [31] under the LG + Γ model. The resulting approximately ML trees with SH-like local supports were examined to identify alignments with aberrant phylogenetic signals that strongly disagreed with well-established monophyletic groups in the eToL, including Opisthokonta, Amoebozoa, Alveolata, Stramenopiles, Rhizaria, Rhodophyta, Chloroplastida, Glaucophyta, Haptophyta, Cryptista, Jakobida, Euglenozoa, Heterolobosea, Diplomonadida, Parabasalia and Malawimonadida. Eleven out of the 351 single-protein alignments were found to violate these criteria and were excluded from further phylogenomic analyses. The remaining 340 single-protein alignments (electronic supplementary material, table S1) were concatenated into a single phylogenomic alignment comprising 132 taxa with 88 397 unambiguously aligned amino acid positions. The coverage for each single-protein alignment is summarized in electronic supplementary material, table S1.

The final alignment comprising 340 proteins from 132 taxa (340-protein alignment) was subjected to the ML analysis using IQ-TREE v. 2.2.2.6 with the LG + Γ + F + C60 model. The robustness of the ML phylogenetic tree was assessed through ultra-fast ML bootstrap approximation with the LG + Γ + F + C60 model (1000 replicates) and a non-parametric ML bootstrap analysis with the LG + Γ + F + C60 + PMSF (posterior mean site frequencies) model (200 replicates) [32]. The ML tree inferred with the LG + Γ + F + C60 model served as the guide tree for the non-parametric bootstrap analysis incorporating PMSF. Additionally, Bayesian phylogenetic analysis was conducted using the CAT + GTR model with PhyloBayes-mpi v. 1.8a. In this analysis, two MCMC runs were conducted for 5000 cycles. After discarding the trees from the first 1250 cycles as burn-in, the consensus tree, including branch lengths and BPPs, was calculated from the remaining trees. In addition, we evaluated each bipartition using AUTOEB v. 1.0.0 [33], in which the two alternative trees on each bipartition were generated by nearest-neighbor-interchange followed by an approximately unbiased (AU) test comparing the original (ML) tree and the two alternative trees. The settings of AUTOEB were set as the default, and the substitution model was the same as the ML phylogenetic analysis of the 340 proteins alignment.

We evaluated the impact of fast-evolving positions within the 340 proteins alignment. Substitution rates for each position were calculated on the ML tree by IQ-TREE v. 2.2.2.6, and four sets of sub-alignments were created from the original alignment by sequentially removing the top 20%, 40%, 60% and 80% fastest-evolving positions. The original alignment and four sub-alignments were individually subjected to the ultra-fast bootstrapping approximation under the LG + Γ + F + C20 model using IQ-TREE v. 2.2.2.6.

### 2.8. Environmental SSU rDNA sequences

To examine the abundance and distribution of *G. oviformis*, we searched for sequences similar to its SSU rDNA sequence in the EukBank global dataset [34] that contains V4 sequences in SSU rDNA from 12 672 samples collected across marine and terrestrial environments. A BLASTn search was conducted using the *G. oviformis* SSU rDNA sequence as a query, and hits with over 90% length of the subject sequence were picked.

## 3. Results

### 3.1. Morphology and ultrastructure of *Glissandra oviformis* n. sp.

**Figure 1.**
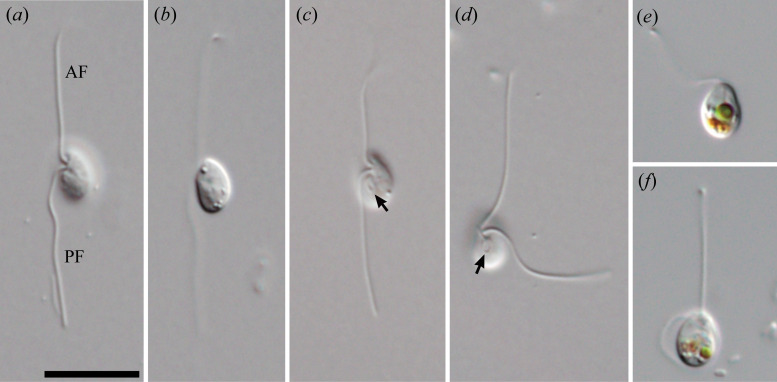
Differential interference contrast micrographs of *Glissandra oviformis* n. sp. (*a,b*) Gliding cells. (*c,d*) Gliding cells showing ventral apertures. (*e,f*) Gliding cells showing food vacuoles. AF, anterior flagellum; PF, posterior flagellum. Arrows indicate ventral apertures. Scale bar = 10 μm.

**Figure 2.**
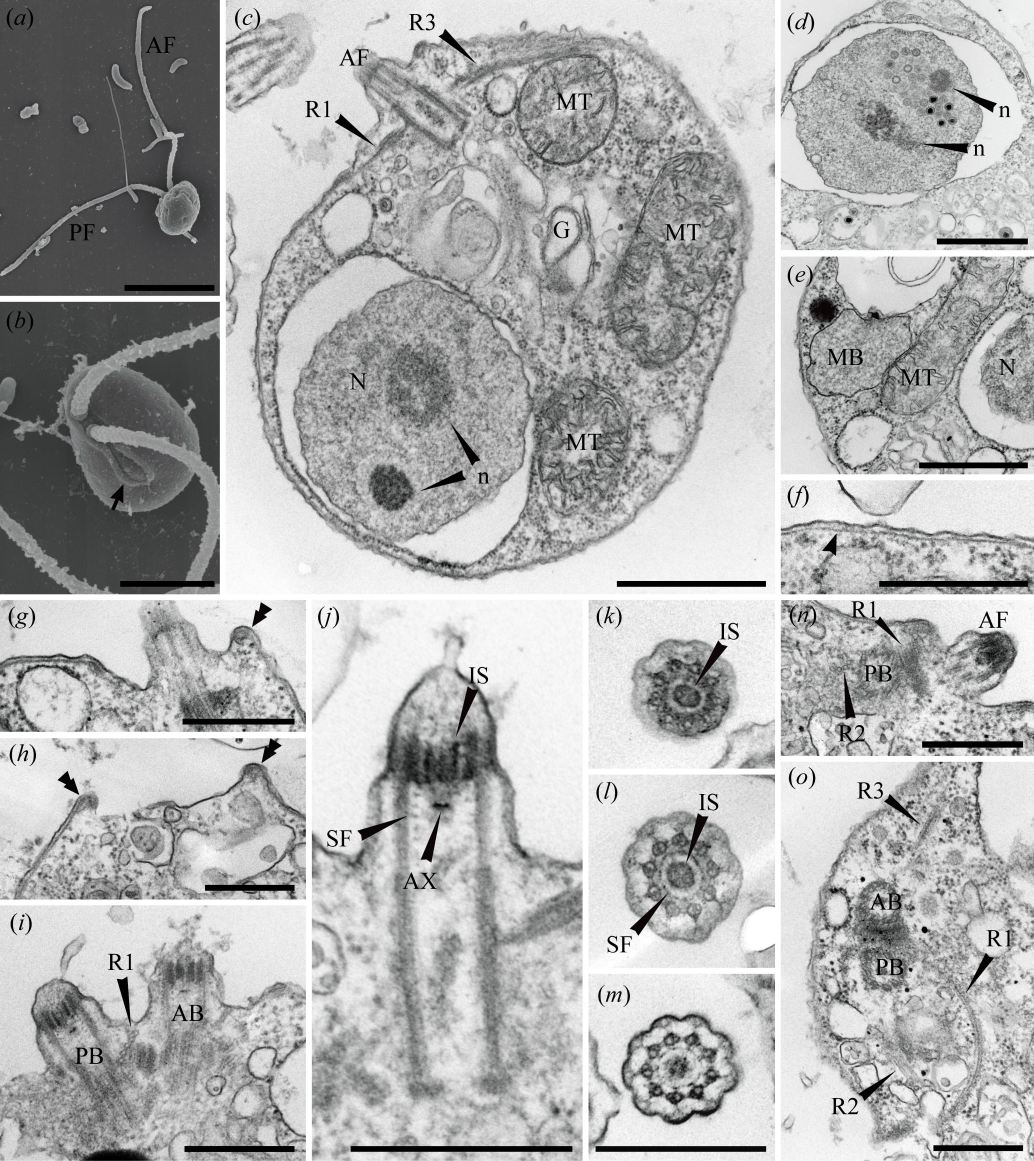
Scanning and transmission electron micrographs of *G. oviformis* n. sp. (*a*) Scanning electron micrograph (SEM) of a whole cell. (*b*) SEM of a flagellar depression and a ventral aperture. (*c*) Transmission electron micrograph (TEM) of the whole cell. (*d*) TEM of a nucleus and virus-like particles in the nucleus. (*e*) TEM of a microbody and a mitochondrion. (*f*) TEM of a pellicle underlying the plasma membrane. (*g*) TEM of inward curling of pellicle at the right edge of the flagellar depression. (*h*) TEM of inward curling of pellicle at the rim of a ventral aperture. (*i*) TEM of the longitudinal section of two basal bodies. (*j*) TEM of the longitudinal section of anterior basal body. (*k*) TEM of a transverse section of a flagellar transitional region at the level of an internal sleeve. (*l*) TEM of a transverse section of a flagellar transitional region at the level between the internal sleeve and a spiral fibre. (*m*) TEM of transverse section of a flagellar transitional region at the level of the spiral fibre. (*n*) TEM of the proximal end of the R2. (*o*) TEM of a transverse section of two basal bodies. AB, anterior basal body; AF, anterior flagellum; AX, axosome; G, Golgi apparatus; IS, internal sleeve; MB, microbody; MT, mitochondrion; N, nucleus; n, nucleolus-like structure; PB, posterior basal body; PF, posterior flagellum; SF, spiral fibre. An arrow indicates ventral aperture. An arrowhead indicates pellicle. Double arrowheads indicate rims of ventral aperture or flagellar depression. Scale bars: (*a*) = 5 μm, (*b–e*) = 1 μm, (*f–h, j, l–o*) = 500 nm.

Cells of *G. oviformis* n. sp. are oval or ovoid, measuring approximately 4.8 (3.7−6.5) μm in length and 3.5 (2.4−5.3) μm in width (*n* = 36) (figure 1). Each cell possesses two flagella of subequal length, 2.5–4 times the cell body length (figure 1*a*,*c*,*d*). These flagella are inserted longitudinally from a depression near the anterior end on the ventral side of the cell (figure 1*a*,*c*,*d*). Cells exhibit gliding motility, with most of both flagella attached to the substrate. During gliding, the anterior flagellum extends straight forward, while its tip detaches from the substrate and moves from anterior to posterior. The posterior flagellum extends posteriorly, attaching to the substrate along its entire length. Cells possess a distinct aperture at the posterior side of the flagellar depression (figure 1*c*,*d*). Some cells contained prey-derived green or orange food vacuoles (figure 1*e*,*f*). In the scanning electron microscopy, the aperture and the flagellar depression are continuous with one another, and their rims were swollen except for the left edge of the flagellar depression (figure 2*a*,*b*). No appendages, such as mastigonemes or scales, were observed on either the flagella or the cell surface (figure 2*a*,*b*).

Transmission electron microscopy showed that *G. oviformis* has a nucleus at the posterior region of the cell (figure 2*c*). The nucleus contains two nucleolus-like structures with different electron densities (figure 2*d*). Virus-like particles covered by envelopes were occasionally observed in the nucleus and cytoplasm (figure 2*d* and electronic supplementary material, figure S1a). Cells have several mitochondrial profiles with lamellar cristae (figure 2*c*,*e*). An inflated Golgi apparatus is situated near the base of the basal bodies (figure 2*c*). In the Golgi cisternae, bridges that link the two closely opposed membranes were observed (electronic supplementary material, figure S1b). These bridges appear to be placed on the same plane (electronic supplementary material, figure S1c). A round microbody was observed in the cell (figure 2*e*). Lipid droplets were occasionally observed in the cytoplasm (electronic supplementary material, figure S1d–g). A single-layered pellicle underlying the plasma membrane, except in the region of the flagellar depression and the aperture, was observed (figure 2*f*). The pellicle exhibits inward curling at the rim of the aperture and the right edge of the flagellar depression (figure 2*g*,*h*). The basal bodies are arranged at an acute angle of approximately 25−40° (figure 2*i*). Both anterior and posterior basal bodies possess dorsally directed fibres (electronic supplementary material, figure S1d–g). A spiral fibre with 4−6 gyres is present in the flagellar transitional region (figure 2*j*,*l*,*m*). In the spiral fibre, a dense plate-shaped axosome is sandwiched by less dense amorphous materials (figure 2*j*). An internal sleeve surrounds the central pair of the axoneme just above the spiral fibre (figure 2*j*–*l*). The basal bodies has three microtubular roots. Following the terminology of Yubuki & Leander [35], each microtubular root was labelled R1, R2 and R3. R1 emerges from the anterior side of the posterior basal body (figure 2*i*). It comprises five microtubules and an electron-dense material associated with its proximal end (figure 2*i*,*n*). R1 initially directs leftward but then curves posteriorly, terminating near the anterior edge of the aperture (figure 2*o*; electronic supplementary material, figure S2). R2 consists of four microtubules arranged in a *U*-shape and emerged from the posterior side of the posterior basal body (figure 2*n*; electronic supplementary material, figures S2c–h and S3g,h). R2 directs towards the anterior edge of the aperture and further extends to line its left edge (figure 2*o*; electronic supplementary material, figure S2). R3 emerges from the anterior side of the anterior basal body, composed of three microtubules (figure 2*c*,*o*; electronic supplementary material, figures S2a and S3a). R3 directs dorsally, passing just beneath the anterior cell surface and lacks secondary microtubules (figure 2*c*,*o*).

### 3.2. Phylogenomic analyses nominated *G. oviformis* as a member of CRuMs

**Figure 3.**
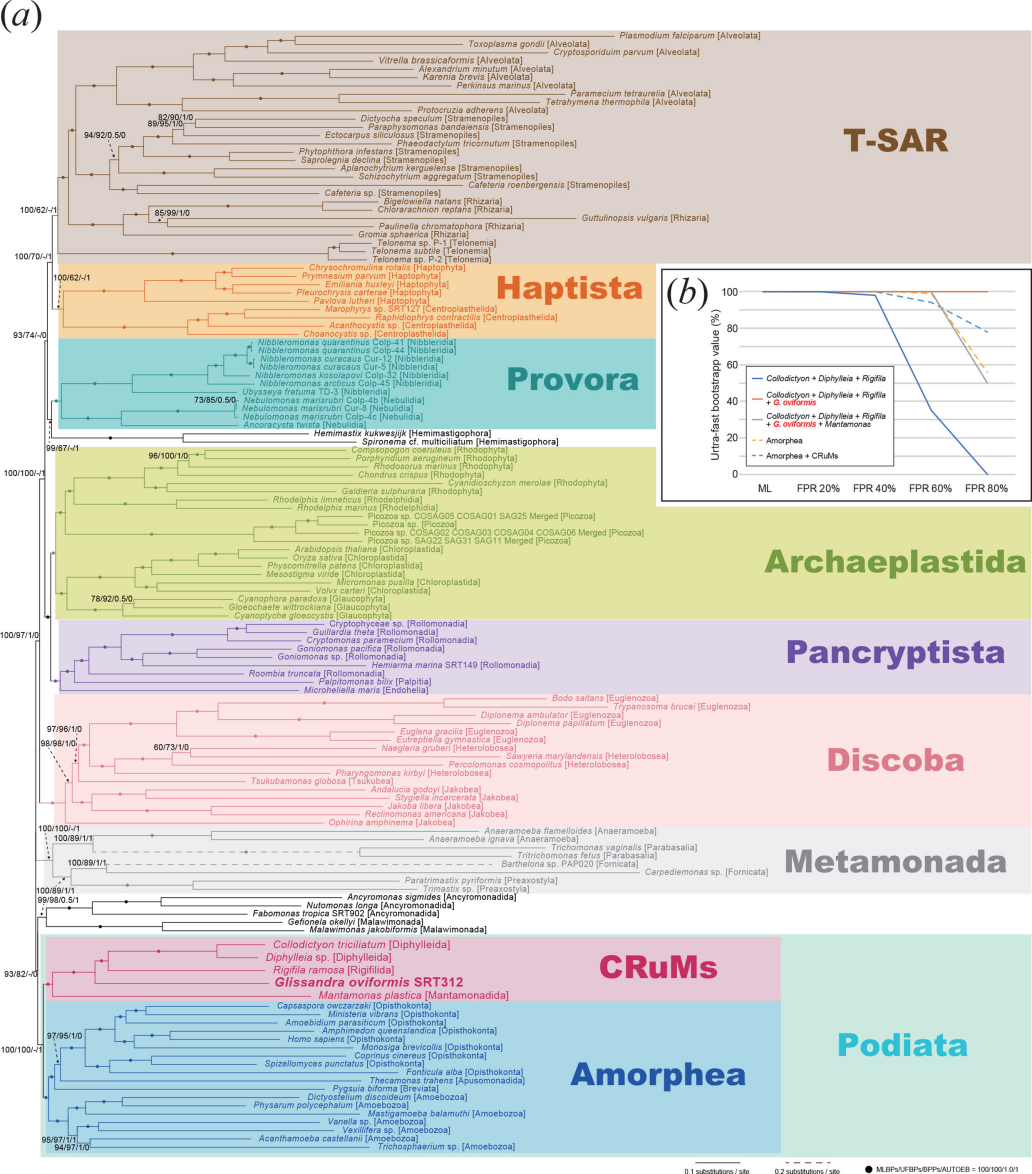
Phylogenomic analyses of a 340-protein alignment. The ML tree was inferred from a 340-protein alignment (comprising 132 taxa and 88 397 amino acid positions) as shown in (*a*). The same alignment was subjected to Bayesian analysis and the resultant BPPs were mapped on the ML tree. The detailed Bayesian tree is provided in electronic supplementary material, figure S4. For each node, MLBPs, ultrafast bootstrap values (UFBPs), Bayesian posterior probabilities (BPPs) and results from AUTOEB analysis are presented. Nodes marked with dots indicate MLBPs = 100%, UFBPs = 100%, BPPs = 1.0 and the calls from AUTOEB as ‘resolved’ (marked by ‘1’). The results from the analyses of the 340-protein alignment processed by fast-evolving position removal (FPR) are presented in (*b*). Ultrafast bootstrap analyses were performed on the original 340-protein alignment and alignments after removing the top 20%, 40%, 60% and 80% of fastest-evolving positions using IQ-TREE 2.2.2.6 under the LG + Γ + F + C20 model. Solid lines in blue, orange and grey indicate UFBPs for the monophyly of (*Collodictyon* + *Diphylleia* + *Rigifila*), (*Collodictyon* + *Diphylleia* + *Rigifila* + *G. oviformis*) and (*Collodictyon* + *Diphylleia* + *Rigifila* + *G. oviformis + Mantamonas*), respectively. Dashed lines in yellow and light blue indicate UFBPs for the monophyly of Amorphea, Amorphea + CRuMs, respectively.

We gathered 190 phylogenetically diverse eukaryotic SSU rDNA sequences to access the phylogenetic position of *G. oviformis*. However, the phylogenetic placement of *G. oviformis* remained unresolved in the SSU rDNA phylogenetic tree. Specifically, *G. oviformis* branched as a sister lineage to Cyanidiaceae, though this bipartition lacked statistical support (an MLBP of 18%, electronic supplementary material, figure S4). To further resolve the phylogenetic position of *G. oviformis*, we performed phylogenomic analyses using a 340-protein alignment from 132 taxa representing all the major eukaryotic groups. The resulting phylogenomic tree robustly supported the monophyly of all the major eukaryotic groups (figure 3*a*). In these analyses, *G. oviformis* was placed within the CRuMs clade, which includes *Mantamonas plastica*, *Collodictyon triciliatum*, *Diphylleia* sp. and *Rigifila ramosa*. The assemblage comprising the previously known members of CRuMs and *G. oviformis* received full statistical support (figure 3*a*). Within this clade, *G. oviformis* branches sister to the clade containing *Rigifila* and two diphylleids, with *M. plastica* branching sister to all other CRuMs members. Each bipartition within this assemblage in the ML tree also received full statistical support and remained consistently supported even after the removal of fast-evolving positions (figure 3*b*). Thus, we conclude that *G. oviformis* is a member of CRuMs and represents a previously overlooked subclade in CRuMs.

It is worth noting the incongruity between the ML and Bayesian analyses. The CRuMs clade enclosing *G. oviformis* is positioned as the sister group to Amorphea (this assemblage is known as Podiata [36], with this relationship being robustly supported in the assessments using the ML method (figure 3*a*). This relationship remains consistent even after excluding fast-evolving positions (figure 3*b*). In contrast, Bayesian phylogenetic analyses placed the clade of parabasalids and anaeramoebas as the sister to Amorphea, thereby failing to recover the Podiata (electronic supplementary material, figure S5). The bipartition uniting Amorphea, parabasalids and anaeramoebas together received an inconclusive BPP of 0.65, suggesting that the precise relationship among Amorphea, CRuMs and the clade of parabasalids and anaeramoebas remains uncertain in Bayesian analysis. In sum, it is too early to conclude the phylogenetic relationship among Amorphea, CRuMs, Malawimonadida and Ancyromonadida (as well as Metamonada) based on the past phylogenomic analyses or that conducted here.

### 3.3. Putative marine habitat of *G. oviformis* and its relatives

A BLASTn search against the Eukbank dataset identified 13 amplicon sequence variants (ASV) aligned to the SSU rDNA of *G. oviformis* in over 90% of their length. One of the 13 ASVs shared 100% identity with the SSU rDNA of *G. oviformis* (electronic supplementary material, table S2). This ASV was assigned the rhodophyta ‘*Porphyra capensis*’ in the Eukbank dataset, but the sequence similarity between the ASV and the SSU rDNA of *P. capensis* deposited in the GenBank database (AY766361) was only 74.4%. Therefore, this ASV originated from *G. oviformis*, not the rhodophyte. We could not conclude the precise origins of the other 12 ASVs, as they did not match the *G. oviformis* sequence or any of the rDNA sequences in the GenBank with high identity (electronic supplementary material, table S2).

The *G. oviformis* ASV was not detected in any terrestrial sample but in 47 samples collected from marine sediment and water columns with a depth range from the surface to 800 m and distributed across the Mediterranean Sea, Indian Ocean, Pacific Ocean and Atlantic Ocean. The *G. oviformis* ASV consists of 5679 reads; most of them (5603 reads) originated from a seawater sample collected from the Indian Ocean and reached over 1% of the total reads. In the other 46 samples, the reads derived from *G. oviformis* occupied less than 0.03% of the total reads (electronic supplementary material, table S3). This low abundance of *G. oviformis* ASV reads can be interpreted as this species being scarce in marine environments on top of its ecological role as a predator.

## 4. Discussion

### 4.1. *Glissandra oviformis* n. sp. as a new species of the genus *Glissandra*

In our light microscopic observation, *G. oviformis* n. sp. showed characteristics consistent with other *Glissandra* species, gliding flagellates possessing anteriorly and posteriorly directed flagella and the anterior to posterior movement of the anterior flagellar tip. *Glissandra* contains two species: *G. innuerende*, the type species and *G. similis* [37,38]. *G. innuerende* resembles *G. oviformis* in attaching both flagella to the substrate and possessing an aperture (as depicted in fig. 8b of [38]) on the ventral side of the cell. However, in *G. innuerende*, the two flagella are inserted side by side, whereas those of *G. oviformis* are inserted vertically. The longitudinal arrangement of flagella in *G. oviformis* was confirmed through our light and electron microscopy, and no variations were observed within the strain. Additionally, there is a slight difference in cell shape, with *G. innuerende* being almost spherical while *G. oviformis* is oval. Another species, *G. similis*, differs from both *G. innuerende* and *G. oviformis*, as it possesses a ventral groove instead of a ventral aperture and attaches to the substrate using the tip of the posterior flagellum [37]. Because *G. oviformis* can be distinguished from the two described species of *Glissandra*, we propose it as a new species of the genus *Glissandra*.

### 4.2. *G. oviforms* reveals character evolution in CRuMs

CRuMs, encompassing three lineages—Diphylleida (Collodictyonida), Rigifillida and *Mantamonas*—has thus far been represented by only 12 species, making it a relatively small group despite the clade being compatible with a supergroup. In this study, our phylogenomic analyses have clarified the phylogenetic position of *G. oviformis* within the CRuMs clade (figure 3*a,b*; electronic supplementary material, figure S5). Combined with the concrete phylogenetic placement of *G. oviformis* within CRuMs, the detailed morphological data from this organism provided the first ground for discussing the character evolution in this clade.

Each of the three CRuMs lineages recognized previously exhibits distinct morphological characteristics. Diphylleida comprises freshwater swimming flagellates with a prominent ventral groove [39,40]. Rigifilida is a group of non-flagellated filose amoebae, including *Rigifila* and *Micronuclearia* [41,42]. *Mantamonas* represents asymmetric, small bacterivorous gliding flagellates consisting of three species, but ultrastructural data for this group are currently unavailable [43,44]. Here, we compare the morphologies and ultrastructures of *G. oviformis* with those of other members of CRuMs and other potentially related taxa to infer the shared characters and synapomorphies of the group.

**Figure 4.**
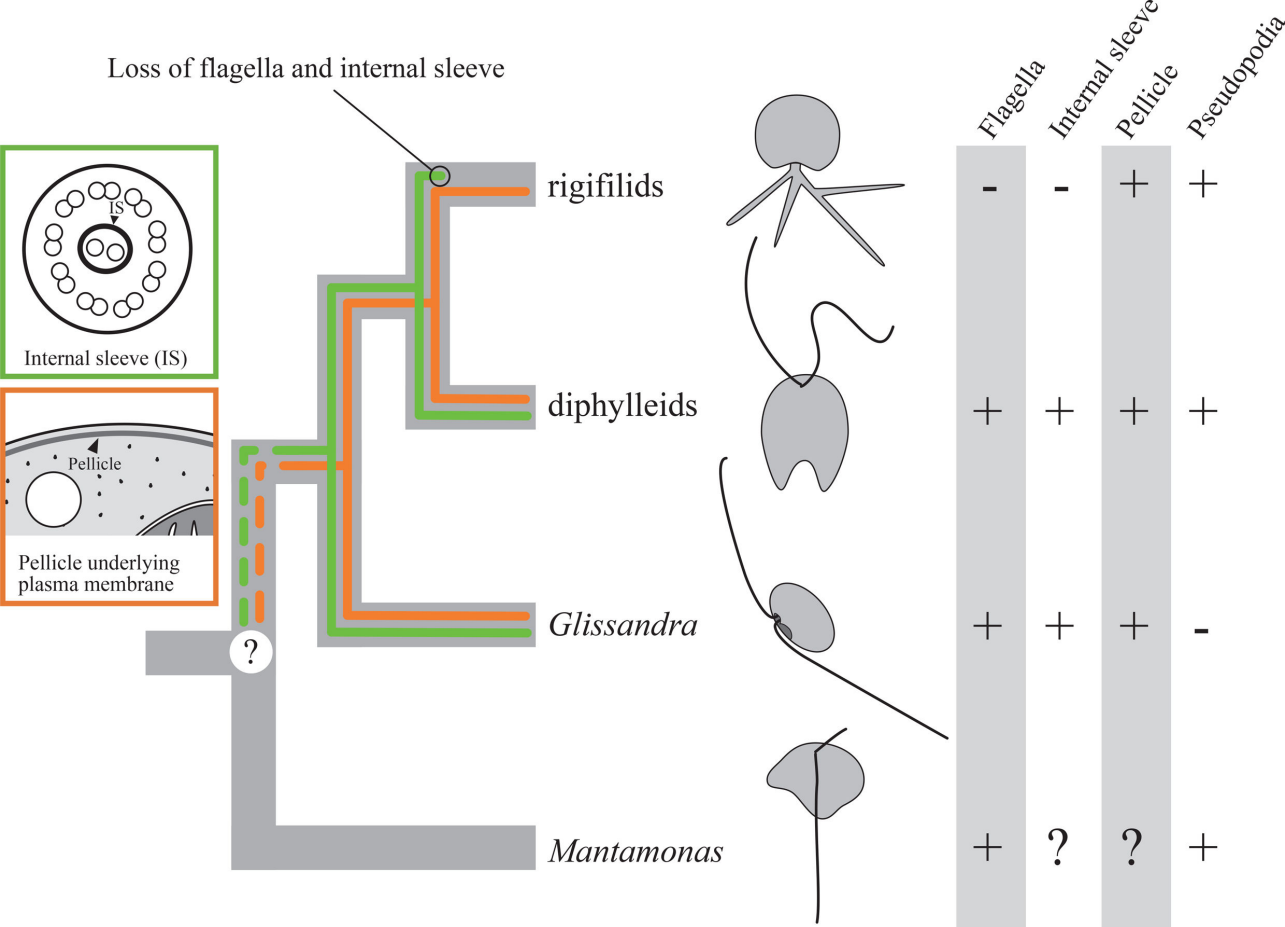
The putative character evolution within CRuMs based on the tree of CRuMs inferred from a 340-protein alignment. Green and orange lines represent the presence of the internal sleeve and the pellicle, respectively.

The presence of pellicles underlying the plasma membrane is observed in the currently recognized members of CRuMs, except for *Mantamonas*, with no ultrastructural data available. Within Rigifilida, single-layered (*Micronuclearia*) or double-layered (*Rigifila*) pellicles support the plasma membrane, with the exception of an aperture through which pseudopodia emerge [41,42]. Although the original ultrastructural studies of diphylleids did not mention a pellicle, Cavalier-Smith [36] pointed out the presence of pellicles in *Collodictyon* and *Diphylleia* (see figure 2e,g,h in [40] and figures 24, 28 and 32 in [45]). The pellicles underlying the plasma membrane appear to extend from the rim of the ventral groove to the dorsal side of the cell. However, it remains unclear whether the entire dorsal surface is covered. Similar to *Micronuclearia* and diphylleids, *G. oviformis* also possesses a single-layered pellicle just beneath the plasma membrane, with exceptions at the flagellar depression and ventral aperture. The widespread occurrence of pellicles suggests that the common ancestor of *Glissandra*, diphylleids and rigifilids possessed this structure (figure 4). We demand the ultrastructural information on *Mantamonas* to clarify whether the pellicle is a shared characteristic among all CRuMs lineages.

Intracellular pellicles are also reported in apusomonads and ancyromonads [46–48]. Ultrastructural studies on ancyromonads reveal a single-layered pellicle underlying the plasma membrane, with exceptions at the base of the flagellar pockets and a channel extending from the posterior flagellar pocket [43,46]. On the other hand, apusomonad pellicle structures vary across studies, ranging from thin, single-layered structures resembling that of *Ancyromonas* [49] to complex, five-layered structures [47,50]. These pellicles underlie the dorsal side of the cell. Apusomonads and ancyromonads were previously classified together in the phylum Apusozoa along with rigifilids based on the presence of pellicles [42,51]. However, recent phylogenomics placed apusomonads within Obazoa, along with Opisthokonta and Breviatea [16,52]. Ancyromonads, on the other hand, remain unclassified but occasionally show a close relationship to Podiata, together with Malawimonadida [14,16,30,52,53]. Our ML phylogenomic tree also suggested that ancyromonads (along with malawimonads) are related to Podiata. However, this topology was not supported by the AU test conducted through AUTOEB and fast-evolving position removal analyses (figure 3*a*,*b*), nor was it reconstructed using the Bayesian method (figure 3*a,b*; electronic supplementary material, figure S5). These morphological features and molecular phylogenies may suggest that pellicles do not originate within CRuMs but is a more ancestral character.

Structures in flagellar transitional regions and fibrillar or microtubular roots associated with the basal bodies have been used as significant characteristics for higher classification of protists [35,54]. While rigifilids are amoebae that lack flagella, and data on the ultrastructure of *Mantamonas* is currently absent, the flagellar apparatus of diphylleids has been extensively studied [39,40,45]. In all genera of diphylleids, a sleeve surrounding the central pair of the axoneme has been reported within the flagellar transitional region [39]. Because we observed the sleeve in *G. oviformis* as well, this feature is the clear synapomorphy for the clade of *Glissandra*, diphylleids and rigifilids, or CRuMs as a whole (figure 4). The flagellar apparatus of diphylleids shares similarities with that of *G. oviformis* in terms of the microtubular roots, which consist exclusively of R1, R2 and R3 (corresponding to lvF, rvF and mL in [45], respectively). Both R1 and R2 are directed posteriorly and R3 is directed dorsally through a position just beneath the anterior surface of the cell. The absence of both the singlet root and R4, which are widely distributed within eukaryotes [35], potentially serve as a taxonomic trait for CRuMs. However, the flagellar apparatus of diphylleids is more complex than that of *G. oviformis* in other aspects. Each microtubular root in diphylleids has an associated electron-dense structure, and R3 possesses well-developed secondary microtubules referred to as a dorsal fan. Additionally, microfibrillar fans are associated with each basal body [39,40,45]. The differences in flagellar apparatus between *G. oviformis* and diphylleids may be related to differences in cell size, mode of movement and feeding behaviour between these two groups.

The shapes of mitochondrial cristae vary among CRuMs. Diphylleids possess tubular cristae, while rigifilids have lamellate (*M. podoventralis*) or irregularly flat (*R. ramose*) cristae [39–42]. *G. oviformis* has lamellar cristae similar to those of *R. ramosa*. This diversity suggests that cristae shape is not a conserved feature within CRuMs. Yabuki *et al.* [42] proposed a potential relationship between rigifilids and diphylleids based on the presence of pseudopodia. Since *Mantamonas* also possesses pseudopodia [44], this feature was likely present in the common ancestor of CRuMs. We did not observe pseudopodia in *G. oviformis*. Therefore, pseudopodia may have been lost in this species or might only appear during specific life stages, such as predation.

### 4.3. Perspectives towards understanding the diversity of CRuMs

It is too naive to assume that the genuine diversity of CRuMs is sufficiently represented by Diphylleida (Collodictyonida), Rigifillida, *Mantamonas* and *Glissandra*. We anticipate that (potentially a number of) as-yet-undescribed members of this clade may remain to be found in natural environments. Despite the diversity of CRuMs being a significant piece of information to picture the eToL, the sequencing analyses based on environmental DNA (eDNA) or cDNAs/genomes amplified from the cells or a single cell isolated from environmental samples are unlikely to be practical tools to identify novel CRuMs members in the future.

Recent advances in metabarcoding have enabled the identification of novel lineages without the extensive time and effort required for establishing laboratory cultures. Marine Stramenopiles (MAST) and marine Alveolata (MALV) are prime examples, significantly contributing to the understanding of the diversity within Stramenopiles and Alveolata lineages [55,56]. In particular, the intra-lineage diversity of organisms that are difficult to cultivate, such as Picozoa, has been substantially expanded through metabarcoding efforts [4,57,58]. Meanwhile, metabarcoding amplifies hyper-variable regions approximately 200−400 bp in length (e.g. the V4 or V9 regions of SSU rDNA) from the organisms present in the environmental sample of interest. When the nucleotide sequence of an amplicon is identical (or very similar) to the SSU rDNA sequence linked to the previously known organism with a cellular identity, we can conclude that the environmental sample examined contained the particular organism. In other words, due to the limited phylogenetic information in the short amplicons, those bearing no similarity to any known SSU rDNA sequences provide little information on their cellular origins. Moreover, there are potentially a large number of organisms that have difficulty in amplifying the regions to be targeted in typical surveys based on eDNA samples, and such organisms may remain undetected (e.g. Euglenozoa and Metamonada [59]). The above-mentioned issues in the experiments based on short amplicons can be overcome partially by amplifying, sequencing and phylogenetically analysing long amplicons (~1 kb), including both variable and conserved regions [60,61]. However, as demonstrated in electronic supplementary material, figure S4, the currently recognized members of CRuMs did not form a single clade in the phylogenetic analysis using (nearly) full-length SSU rDNA sequences. Even closely related species, such as *Diphylleia* and *Collodictyon*, form distinct clades in the SSU rDNA phylogeny. Thus, phylogenetic analyses of full-length SSU rDNA sequences are most likely insufficient to identify novel members of CRuMs. Only phylogenomic analyses based on high-quality transcriptome data unveiled novel members of CRuMs and their internal relationships with confidence.

We can now obtain transcriptome and genome data from a small number of cells (even a single cell) isolated from environmental samples, and such large-scale sequence data have been regarded as an efficient method for resolving various subtrees of the eToL [62]. However, it is difficult to tell which cell is a novel CRuMs member under a microscope, as the cell morphology varies largely among the currently known members in this clade. Thus, the survey explicitly targeting novel CRuMs members, followed by single cell-based, large-scale sequencing, is likely infeasible. Unfortunately, there is no experimental shortcut for the studies exploring the diversity and evolution of CRuMs. We must keep searching for previously undescribed eukaryotes in various types of natural environments to encounter novel organisms belonging to CRuMs in the future. It is also possible that, as *Glissandra*, novel CRuMs members might be overlooked among the previously described PUPA.

### 4.4. Taxonomic summary

CRuMs

Order Glissandrida ord. nov.

Diagnosis: Heterotrophic biflagellates. Flagellar transitional region includes the sleeve that surrounds the central pair of flagellar microtubules. Single-layered pellicle that underlying the plasma membrane except for a flagellar insertion and a ventral aperture. Mitochondrial cristae with irregularly flat shape.

Family Glissandridae fam. nov.

Diagnosis: Same as order.

Type genus: *Glissandra*

ZooBank LSID: zoobank.org:act:F672EB09-AD50-41A5-9B98-395AA082701E.

Genus *Glissandra*

Emended diagnosis: Gliding biflagellates. Anterior flagellum directed anteriorly and the posterior flagellum directed posteriorly in gliding cell. Only the tip of the anterior flagellum moves from anterior to posterior during gliding. Ventral aperture or ventral groove present.

ZooBank LSID: zoobank.org:act:84C012D3-A6C2-44BE-9E09-68B8A28EE65F.

*Glissandra oviformis* n. sp.

Diagnosis: Cells are oval or ovoid, 3.7−6.5 μm in length and 2.4−5.3 μm in width. Two subequal flagella of 2.5−4 times the cell length inserted vertically from a subapical depression at ventral side of the cell. Both flagella lie against the substrate in gliding cells.

Hapantotype: One microscope slide (TNS AL-66024s) deposited in the herbarium of the National Museum of Nature and Science (TNS), Tsukuba, Japan.

Paratype: One EM block (TNS AL-66024tb) deposited in the TNS. These cells are derived from the same sample as the hapantotype.

DNA sequence: Small subunit ribosomal RNA gene, LC868286.

Type locality: A sample of seaweed (*Halimeda* sp.) in Coral Lake, the Republic of Palau (latitude = 7.2510°N, longitude = 134.3738°E).

Collection date: 1 November 2013.

Etymology: The specific epithet *‘oviformis’* (egg-shaped) refers to the cell shape of this organism.

ZooBank LSID: urn:lsid:zoobank.org:act:7F61F697-D9F6-4901-B21E-E94426AE4C40.

## Data Availability

The SSU rDNA sequence of *Glissandra oviformis* n. sp. was deposited in the DDBJ database under accession no. LC868286. The transcriptome data of *G. oviformis* was deposited in the DDBJ Sequence Archive under Bioproject PRJDB20542. The assembled transcriptomes of *G. oviformis* and phylogenetic alignments analysed in this study are available from supplemental data. The assembled transcriptomes of *G. oviformis* and phylogenetic alignments analysed in this study are deposited in Dryad [63]. Supplementary material is available online [64].
